# Medical History from the Earliest Times

**Published:** 1892-04-23

**Authors:** E. T. Withington


### Medical History from the Earliest Times.

By E. T. Withington, M.A., M.B. Oxon.

### V.—MEDICINE IN ANCIENT EGYPT.

According to Clement of Alexandria, the knowledge of the Egyptians was contained in forty-two sacred books attributed to the god Hermes (Thoth), of which the last six were medical, and treated of anatomy, diseases, instruments, drugs, and affections of the eye, and of women respectively. Manetho says that Athothis, second King of Egypt, wrote on anatomy, a story probably due to the resemblance of the names Athothis and Thoth, and Professor Ebers considers that his papyrus is nothing less than the Hermetic book on drugs. One hardly ventures to question so high an authority, but so far as can be judged from translations, the work seems to be rather a compilation than a sacred book. The chapters are of various age and authorship, and deal with other matters besides drugs, as, for instance, the doctrine of the heart and vessels, which is called the "secret of the physician," while the prescriptions given are by no means considered to be all of equal value. As to the origin of the work, it commences by saying, "I came forth from On" (Heliopolis), but in the next line we have "I came forth from Sais," as if the compiler had made extracts from the medical libraries of several temples. All are agreed that charms and incantations are much less prominent in this than in later medical papyri. Galen, as we shall shortly see, was by no means above ordinary superstition, but the sacred books of the Egyptians went too far even for him, and he characterises them briefly as "all nonsense," a term which he would hardly have applied to the Ebers papyrus. Jamblichus tells us that the Egyptian philosophical books had become much altered in the hands of the Alexandrine Gnostics and Neoplatonists, and one is tempted to think that the same was the case with the medical works, and that the books which Clement saw, and Galen read, bore more resemblance to those fragments of theosophy which have come down to us under the name of Hermes Trismagistus, than to the great Leipsic papyrus.

The other medical papyri are of little importance. One, at Berlin, somewhat resembles that of Ebers, but is smaller and of later date. It contains seventy prescriptions, no less than twenty-eight being enemata, a form of medication the origin of which was universally ascribed to the Egyptians. The continuance, or, perhaps, the revival of the demonic theory is indicated by the fact that the remedies are said to "attack," "repulse," or "destroy" the disease, the expressions "cure" or "alleviate" being rarely used. About the time this papyrus was written occurred the famous trial of the conspirators against Rameses III. (B.C. 1,200), one of whom, a high official, is charged with stealing a book on witchcraft from the royal library, and introducing waxen images made according to its directions, into the palace, with intent to cause disease and death. The later writings are almost entirely given over to charms and love philtres.

Whatever we may think of Athothis, one Egyptian monarch certainly wrote on medicine—Nachepsus of Sais (B.C. 700), who is quoted both by Pliny and Galen. The latter tells us that this king was the first to observe the marvellous virtues of green jasper, a stone which, when engraved with "a dragon with rays," and hung round the neck, is a sovereign cure for digestive disorders. Galen says he has made numerous experiments with these stones, and finds them no less effectual without the dragon. Some very beautiful examples of the engraved amulets may be seen among the Gnostic gems in the British Museum, where the Greek inscriptions call the dragon "Chnoumis, the Destroyer of Demons."

The Egyptian physicians presided over the process of embalming (Genesis 50, 2), but they seem to have entrusted the practical part to the temple servants, and as there is no evidence that the custom in any way furthered medical or anatomical knowledge, we need not here repeat the descriptions of Herodotus.

The surgical division of the healing art appears to have been much neglected, as has always been the case when medicine was in the hands of priests. The Bulak Museum contains a fractured thigh-bone, in which the fragments overlap more than two inches, and Mariette has met with many similar cases, which, he says, have given him a very poor idea of Egyptian surgery. The stories of pictorial representations of amputation, and of teeth stopped with gold found in mummies, have not been confirmed by later investigators.

Up to a recent period our knowledge of Egyptian medicine was gathered solely from scattered passages in Greek writers, some of which have already been noticed. The early Greeks looked with admiring wonder on the ancient civilization of Egypt, and observing the strict dietetic rules of its inhabitants, who took medicines periodically even when in health, concluded that every Egyptian was a physician, and possessed of marvellous skill. In later times Herodotus and the prophet Jeremiah notice the numerous physicians, and the multitude of medicines, and the former writer tells us that each one devoted himself to some special disorder, a system only possible in very populous localities, or where the practitioner is not immediately dependent upon his patients. Both these conditions were present in Egypt, where, in the reign of Amasis, twenty thousand towns and villages filled the valley of the Nile, and where the physicians shared in the general income of the temples, to which the contributions of their patients were probably added.

Some of the stories told by late writers, though of rather doubtful authenticity, are worth repeating. Thus, the people of the Delta are said to have found the squill (Scilla maritima) so useful in dropsy that they erected a temple in its honour; and we are told that Euripides, the poet, was recommended by the priests to take a course of sea bathing, and was much benefited thereby.

But the most remarkable thing about Egyptian medicine is its non-progressive character. More than a thousand years before Hippocrates we find a knowledge of anatomy and physiology quite equal to that of the Father of Medicine, together with a copious and varied materia medica, comprising mineral as well as vegetable remedies; yet when, in the sixth century B.C., Egyptian physicians come in contact with a

Greek, they prove hopelessly his inferiors, and, as we shall see, owe their very lives to his skill. The Greek physicians of Alexandria seem to have learnt nothing from the Egyptians, and Galen mentions their medical writings with contempt. Let us try to find some causes for this remarkable failure.

It used to be thought that Egypt, for some mysterious reason, stood still for forty centuries, and that it made little difference to an ancient Egyptian whether he was born 4,000 or 400 years B.C.; but we now know that the nation underwent many changes, social and intellectual, no less than political, during this vast period. Had that ancient practitioner, Sekhet'enanch, returned to life in the days of Moses, he would have found his language scarcely understood, his dress worn only by statues of the gods and Pharaohs, and the state of society entirely altered. What changes he would have noticed in his own art we, unfortunately, do not know; though, in all probability, medicine had already assumed a more formal and priestly character.

But, while this predominance of the priesthood was doubtless a chief cause of the failure of the early promise of Egyptian medicine, there were others almost as important. The Egyptians were essentially a matter-of-fact race—types of those practical people of whom it has been well said that they practise the errors of their forefathers. Learning, indeed, is often praised by ancient writers, but only as a means to some material benefit; nor is there a trace of that love of knowledge for its own sake, without which no great progress in science has ever been made. "My son, apply thyself to learning that thou mayst be a scribe," writes an old Egyptian. "The people are heavily-laden asses, but the scribe is the driver. The scribe is never hungry; he sits at Pharaoh's table, and his belly is filled by reason of his wisdom." The Greek was the very reverse of this, and ever ready to speculate upon or investigate any subject for the mere pleasure of doing so. Science to him was a majestic goddess, a clear-eyed Pallas Athene; to the Egyptian she was a domestic cow, good only for what could be got out of her.

Again, the learned Egyptian was, before all things, a scribe or writer. He had invented no less than three forms of that art, an achievement of which he was justly proud, and one of the commonest figures in representations of Egyptian life is the scribe with his ink-pot taking notes. It is easy to see how this might lead to excessive reverence for what was written, in so much that "book" and "science" came to be expressed in Egyptian by the same word. The Greek, on the contrary, was a man of speech and argument. Plato tells us that a physician did not prescribe for a free man till he had persuaded him, and though the custom of a patient arguing with his doctor has obvious disadvantages, it tends at least to keep the latter from sinking into a sleepy routine.

